# Comparison of multipoint linkage analyses for quantitative traits in the CEPH data: parametric LOD scores, variance components LOD scores, and Bayes factors

**DOI:** 10.1186/1753-6561-1-s1-s93

**Published:** 2007-12-18

**Authors:** Yun Ju Sung, Yanming Di, Audrey Q Fu, Joseph H Rothstein, Weiva Sieh, Liping Tong, Elizabeth A Thompson, Ellen M Wijsman

**Affiliations:** 1 Division of Medical Genetics, Department of Medicine, University of Washington, Box 357720, Seattle, Washington 98195-7720, USA; 2 Department of Statistics, University of Washington, Box 354322, Seattle, Washington 98195-4322, USA; 3 Department of Biostatistics, University of Washington, Box 357232, Seattle, Washington 98195-7232, USA

## Abstract

We performed multipoint linkage analyses with multiple programs and models for several gene expression traits in the Centre d'Etude du Polymorphisme Humain families. All analyses provided consistent results for both peak location and shape. Variance-components (VC) analysis gave wider peaks and Bayes factors gave fewer peaks. Among programs from the MORGAN package, lm_multiple performed better than lm_markers, resulting in less Markov-chain Monte Carlo (MCMC) variability between runs, and the program lm_twoqtl provided higher LOD scores by also including either a polygenic component or an additional quantitative trait locus.

## Background

Our aims were 1) to compare results from several multipoint linkage analysis programs that are available for quantitative traits and 2) to investigate the performance of MCMC-based programs on the GAW15 expression data in 14 three-generation CEPH families genotyped for clustered SNP markers [1]. We used three recently developed programs in the MORGAN package [2]: lm_markers, lm_multiple, and lm_twoqtl. These programs provide MCMC-based parametric LOD score analysis, the first two with a one-QTL (1Q) model and the last with more complex models, including a second linked (2Q) or unlinked (UQ) QTL and/or a polygenic component (P). In addition, we used Loki [3] for Bayesian oligogenic analysis and Merlin [4] for VC analysis. These analyses cover most approaches that fully use quantitative trait data from three-generation pedigrees.

## Methods

### Phenotypes used

For 62 traits previously reported to show evidence of linkage [5,6], we performed genome-wide VC analysis and obtained the maximum likelihood estimate (MLE) of heritability (*h*^2^). We chose six traits that showed high VC LOD scores and *h*^2 ^≥ 0.31: *CHI3L2*, *GSTM1*, *PSPH*, *VAMP8*, *PPAT*, and *TM7SF3*. The first two of these had only a single peak with VC LOD > 3, representing potentially simple traits, and the latter four had multiple peaks, representing potentially complex traits. For these six traits, we performed Bayesian oligogenic joint segregation and linkage analyses using Loki and parametric LOD score analysis with a 1Q model using lm_markers and lm_multiple. For the first four traits only, we also performed parametric LOD score analysis with more complex models using lm_twoqtl.

### Genetic map and marker data

We used the Rutgers map [7] for linkage analysis. We converted Kosambi map positions to Haldane map positions for analysis, although for ease of comparison with other GAW contributions we present all results on a Kosambi scale. We also constructed a jittered map by adding 0.01 cM between markers with identical positions on this map. We excluded sex chromosomes and used the sex-averaged jittered map for all our linkage analyses because neither MORGAN nor Loki allows multiple markers at the same position. For the VC analysis, we also used the nonjittered map as a comparison. We used Merlin to identify all Mendelian-inconsistent genotypes (69 marker-family combinations) and any obligate recombinations within each cluster (166 cluster-family, or 508 marker-family combinations), where a cluster is defined as a set of markers that have the same Rutgers map position. We coded these markers as missing genotypes in all members of the families with an apparent error.

### Segregation and linkage analyses

For the 62 traits, we performed genome-wide VC linkage analysis with Merlin for both the jittered and original nonjittered maps. VC LOD scores were computed only at the marker positions. We also obtained MLEs of *h*^2 ^for these 62 traits with a VC polygenic model [8]. Using Merlin, we obtained MLEs of marker allele frequencies, which we used in all linkage analyses.

For the six traits, we performed Bayesian oligogenic segregation analysis and oligogenic joint segregation and linkage analysis using Loki. For segregation analysis, we used every fourth iteration in a 50 k iteration run to estimate QTL models. For linkage analysis, we used every fourth iteration in a 999 k iteration run to compute Bayes factors for presence versus absence of a QTL in each 2-cM bin. We used QTL models estimated from Bayesian segregation analysis in all our LOD score analyses.

We recently developed three programs in MORGAN: lm_markers, lm_multiple, and lm_twoqtl. The first two programs compute LOD scores for the 1Q model, and lm_twoqtl computes LOD scores for more complex models [9]. In addition to its MCMC-based approach, lm_markers now can also provide exact computation of LOD scores for small pedigrees with many markers. No other programs provide parametric LOD scores for quantitative traits with many markers. The program lm_multiple differs from lm_markers only in that, instead of updating only one meiosis at a time, it uses an improved sampler that simultaneously updates either a randomly chosen subset of up to eight meioses or a possibly larger subset of meioses in closely related individuals, such as siblings [10]. This multiple-meiosis updating can improve estimates of LOD scores, particularly for data with large sibships. Finally, lm_twoqtl provides LOD scores with models that include additional linked or unlinked QTLs and a polygenic component. Incorporating better modeling of complex traits into linkage analysis can provide higher LOD scores and better localization for complex traits [9].

We performed parametric linkage analysis using these three MORGAN programs. For the six traits, we obtained ten estimates of LOD scores using MCMC and both lm_markers (3 k and 30 k scans) and lm_multiple (3 k scans), to compare their performance. For comparison, we also computed exact LOD scores for the 1Q model, also using lm_markers. Parameter values for the trait model were almost identical to those for the mixed model in Table 1, except for using σ^2^(a) + σ^2^(e) as the environmental variance. For the first four traits, we also used lm_twoqtl with one linked plus one unlinked QTL (1Q + UQ) and one QTL plus a polygenic component (1Q + P) models. In addition, for *VAMP8*, we used lm_twoqtl with a two-linked-QTL (2Q) model. For the first three traits, the secondary QTL model was from oligogenic segregation analysis, whereas for *VAMP6*, the secondary QTL model was the same as the first QTL model. LOD scores at the marker positions as well as midway between two markers were evaluated for all MORGAN programs. We obtained initial starting configurations by using sequential imputation for all MORGAN programs and the locus sampler for Loki. Burn-in iterations were 150 for all MORGAN programs and 1000 for Loki. We used a 50:50 ratio of locus to meiosis sampler for all MCMC-based analyses. For lm_multiple, the probabilities for updating meioses from random subsets, individuals, full sibships, and full three-generation families were 0.2, 0.3, 0.3, and 0.2. For lm_twoqtl, we used every tenth scan in a 30 k scan run for computing LOD scores. For lm_markers and lm_multiple, we used every scan.

**Table 1:** Oligogenic segregation analysis results

	Trait	Transcript	P(A)	μ(AA)	μ(Aa)	μ(aa)	σ^2^(q)	σ^2^(a)	σ^2^(e)	*h*^2 ^Loki	*h*^2 ^MLE
1	CHI3L2	213060_s_at	0.56	7.98	9.84	10.51	0.96	0.24	0.22	0.80	0.69
2	GSTM1	204550_x_at	0.77	8.01	9.17	9.50	0.35	0.03	0.15	0.70	0.68
3	PSPH	205048_s_at	0.89	6.43	8.88	9.51	1.02	0.55	0.12	0.85	0.64
4	VAMP8	202546_at	0.28	10.20	10.36	10.69	0.03	0.02	0.07	0.38	0.38
5	PPAT	209433_s_at	0.21	8.73	9.59	9.70	0.04	0.07	0.08	0.55	0.33
6	TM7SF3	217974_at	0.19	5.20	6.82	6.96	0.11	0.17	0.20	0.56	0.31

P(A), frequency of allele A; μ(AA), phenotypic mean of genotype AA; σ^2^(q), variance due to the major QTL; σ^2^(a), polygenic variance; σ^2^(e), environmental variance; *h*^2^, heritability

## Results

### VC LOD scores and heritabilities for the 62 traits

Of the 62 traits, 24 had a VC LOD score ≥ 3, with *h*^2 ^ranging from 0.13 to 0.86. Five traits had a maximum VC LOD score < 1, with *h*^2 ^ranging from 0 to 0.11. Most traits had only a single peak in the genome with VC LOD ≥ 3, suggesting a simple mode of inheritance. Two traits (*PSPH *and *DDX17*) had three peaks with VC LOD ≥ 3, and three traits (*PPAT*, *HSD17B12*, *TUBG1*) had two peaks with VC LOD ≥ 3. The jittered and nonjittered maps yielded virtually identical VC LOD scores, except for *VAMP8 *on chr 2, where the largest peak was slightly narrower with the nonjittered map.

We chose the six traits *CHI3L2*, *GSTM1*, *PPAT*, *PSPH*, *TM7SF3*, and *VAMP8 *for further analysis. The actual locations of these genes were at the maximum VC LOD scores (*CHI3L2*, *GSTM1*, *PSPH*), 10 cM away (*VAMP8*), or 25 cM away (*PPAT*). Bayesian oligogenic segregation analysis for these traits provided posterior mean numbers of QTLs ranging from 2 to 3.5. Estimation of the primary QTL model was relatively straightforward (Table 1), whereas the secondary or weaker QTL models were less obvious. Heritabilities estimated from Bayesian oligogenic segregation analysis were sometimes higher than MLEs of *h*^2 ^obtained from a VC polygenic model. This is not surprising because VC analysis with Merlin uses only additive genetic variance, thus providing only narrow-sense heritabilities, whereas Loki allows for dominance effects, thus providing larger broad-sense heritabilities.

### Bayes factors using an oligogenic model for the 6 traits

**Figure 1:**
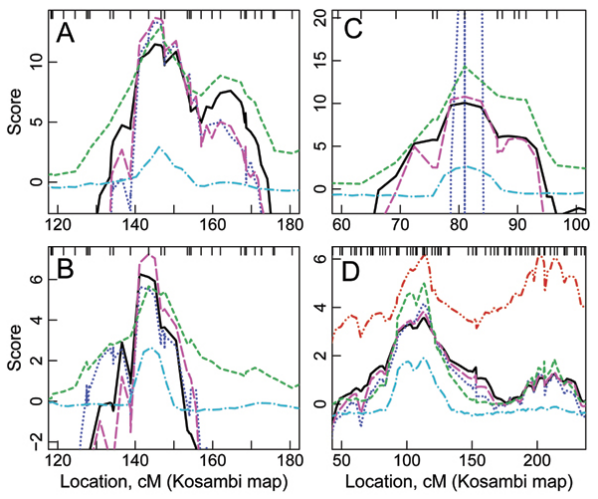
**Linkage analyses of 4 traits**. A, *CHI3L2 *on chr 1. B, *GSTM1 *on chr 1. C, *PSPH *on chr 7. D, *VAMP8 *on chr 2. One linked QTL plus polygenic (magenta, long-dashed), one linked QTL plus one unlinked QTL (blue, dotted), one QTL (black, solid), VC (green, short-dashed), log 10 of Bayes factors (cyan, dot-dashed), and two linked QTLs (red, dot-dot-dashed).

Bayes factors generally matched the VC LOD scores in both peak location and general shape (Figure 1, Table 2), with two minor differences. First, Bayes factors provided much narrower peaks than did VC LOD scores. Second, Bayes factors did not provide several modest peaks that were obtained with VC analysis. For *PSPH*, Bayes factors did not provide evidence of linkage on chr 2, whereas VC LOD scores provided bimodal peaks with VC LODs of 2.6 and 2.8. Also, Bayes factors did not confirm a secondary peak obtained by VC analysis on chr 8 for *PSPH *and chr 2 for *VAMP8*. The primary QTL model estimated from segregation analysis almost always appeared on the chromosomes with the strongest linkage signals. The traits with support for linkage to more than one chr are: *PSPH *with a strong signal on chr 7 (Fig. 1C) and a modest signal on chr 8, *TM7SF3 *with moderate signals on both chr 2 and chr 12, and *VAMP8 *with a strong signal on chr 2 (Fig. 1D) and a weaker signal on chr 4.

**Table 2:** Highest LOD score or log (Bayes factor) and run time (in minutes)

			*CHI3L2 *147 cM (chr 1)^a^		*GSTM1 *142 cM (chr 1)		*PSPH *80 cM (chr 7)		*VAMP8 *113 cM (chr 2)	
						
Model	Program	Scans	Stat^b^	Time	Stat	Time	Stat	Time	Stat	Time
1Q	Exact	NA	11.5	1229	6.3	1234	10.1	470	3.6	1044
	lm_multiple	3 k	11.3–11.5	44	6.2–6.3	45	9.9–10.1	33	3.6–3.6	43
	lm_markers	3 k	10.7–11.6	21	5.7–6.3	21	8.1–10.3	13	3.5–3.6	20
	lm_markers	30 k	10.6–11.6	177	5.7–6.3	168	8.1–10.1	110	3.2–3.6	153
1Q + P	lm_twoqtl	30 k	13.7	563	7.2	604	10.8	401	3.8	535
1Q + UQ	lm_twoqtl	3 k	13.4	3568	5.6	816	40.4	542	4.1	808
VC	Merlin	NA	13	2	5.7	2	14.3	1	5	2
Bayesian	Loki	999 k	2.9	707	2.6	700	2.6	504	1.9	513

^a^peak position (± 1 cM) from all analyses and gene location except *VAMP8 *(120–123 cM).

^b^MORGAN and VC programs, the statistic (stat) is the LOD score with range (min and max) over 10 runs and time is the median of 10 runs for MCMC programs; Loki, stat is the log_10 _(Bayes factor) for one run.

### LOD scores using a one-QTL model for the six traits

Model-based LOD scores matched VC LOD scores in both peak location and general shape (Fig. 1, Table 2). The only minor difference was that the model-based LOD score did not provide a third peak between the two peaks that the VC LOD score provided for *TM7SF3 *on chr 12. For most traits several of the 14 pedigrees were almost uninformative for linkage, the model giving negligible probability that the QTL was segregating in the pedigree (Table 3). For *PSPH*, the low trait allele frequency led to 9 of the 14 pedigrees being uninformative.

**Table 3:** Exact LOD scores by family at chromosomal locations with the highest overall LOD score

			Pedigree														
			
Trait	Chr	cM	1	2	3	4	5	6	7	8	9	10	11	12	13	14	All
*CHI3L2*	1	147	0.6	0.76	2.34	2.34	-0.03	1.84	-0.72	1.09	1.68	2.02	-0.62	-0.05	0.79	-0.56	11.48
*GSTM1*	1	142	0	1.67	-0.01	1.4	0.42	0	0	0	0	-1.09	1.16	1.48	0.34	0.89	6.26
*PSPH*	7	80	2.34	0	0	2.03	0	0	0	2.01	0	0	2.03	0	0	1.64	10.05
*VAMP8*	2	113	0	0.08	0.31	-0.06	0.62	0.33	0.47	-0.17	1.01	0.38	0.21	0.49	-0.03	-0.08	3.56
*PPAT*	4	78	0.1	0.01	0.31	0.01	1.23	0	0.01	0	0.14	0.01	-0.11	0	0.02	1.49	3.22
*TM7SF3*	12	55	0.01	0.33	0	0.09	0	0.02	0	0	0.04	1.02	0.03	0	0	0	1.54

**Figure 2:**
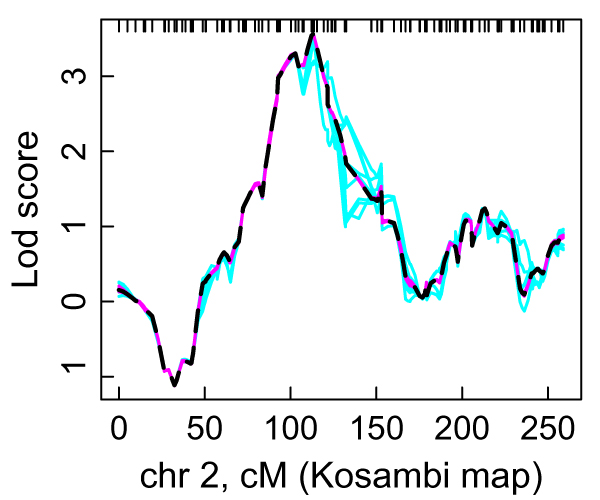
**Linkage analyses of *VAMP8 *on chromosome 2 using 1Q model**. 10 lm_markers runs with 30 k scans (cyan, solid), 10 lm_multiple runs with 3 k scans (magenta, solid), and exact run (black, medium-dashed).

For all six traits, lm_multiple runs with 3 k scans provided better results than lm_markers runs with 30 k scans. Computation time for 3 k scans with lm_multiple was about one-third that of 30 k scans with lm_markers (Table 2). In particular, for *VAMP8*, all 10 lm_multiple runs were an almost perfect match to the exact LOD scores, whereas lm_markers runs with 30 k scans showed moderate run-to-run variation (Fig. 2). For all six traits, lm_multiple showed the smallest run-to-run variation of the LOD scores at the peak (Table 2) as well as elsewhere on the chromosome. Runs of lm_markers with 3 k scans were not much different and showed only slightly more variability from runs with 30 k scans.

### LOD scores using more complex models for the four traits

More complex trait models lead to higher LOD scores than the 1Q model (Table 2). For *GSTM1*, the 1Q + P model provided the highest LOD scores (Fig. 1B), while for *CHI3L2 *and *VAMP8*, LOD scores for 1Q + UQ and 1Q + P models were almost identical (Fig. 1A, D). For *CHI3L2*, the model labeled as 1Q + UQ in Table 2 actually included a polygenic component, i.e., 1Q + UQ + P, which increased the run time significantly. In contrast, for *PSPH*, the 1Q + UQ model provided strange results, with LOD scores ranging from less than -3000 to 40 (Fig. 1C). This may be due to inaccurate estimation of the secondary QTL model: the combined genetic variance from the two QTLs exceeded the total genetic variance obtained from segregation analysis. For *VAMP8*, the 2Q model provided two peaks, of equal magnitude (Fig. 1D), resulting from the identical model for both QTLs.

## Discussion

We performed several multipoint linkage analyses for quantitative traits: VC, Bayesian oligogenic, and parametric LOD score linkage analysis with 1Q, 1Q + P, 1Q + UQ, and 2Q models. We found that all of these analyses provided similar inferences about peak location and shape, with some advantage to using the 1Q + P and 1Q + UQ models over the 1Q model. Use of parametric LOD scores also provided insights into genetic heterogeneity of the traits, which was considerable. However, models for QTLs other than the primary QTL were difficult to estimate with the Bayesian approach for these gene expression traits, suggesting the need for better segregation analysis tools for estimating parameters of complex trait models.

We were able to obtain reliable results for analysis with clustered SNPs with several newly-developed MCMC programs in MORGAN. We found that lm_multiple provided better estimates of LOD scores than lm_markers with fewer scans in less time although, in general, both programs performed well with only minor differences in the variability between runs. The MCMC performance obtained here is improved relative to our results for GAW14 [11]. Factors in this improvement likely include the use of sequential imputation to obtain starting configurations [12], less missing data, and different SNP marker maps, in addition to improved algorithms and software. Finally, although our goal here was to compare our developing MCMC-based methods, we advocate use of exact computation when this is practical. On small pedigrees, such as those used here, exact analysis with a 1Q model and lm_markers or with VC methods may be best initially since this is faster than MCMC analysis. Further analyses may use lm_twoqtl, if the evidence warrants it. However, on larger pedigrees, exact multipoint computation may not be possible, in which case these MCMC options are a viable and practical alternative.

## Conclusion

We showed that MCMC-based programs from the MORGAN package provide accurate LOD scores for quantitative traits with SNP markers. The program lm_multiple gives more accurate results than lm_markers, and the program lm_twoqtl expands the trait models to include two loci plus a possible polygenic component.

## List of Abbreviations

1Q: One QTL

1Q + P: One QTL plus a polygenic component

1Q + UQ: One linked QTL plus one unlinked QTL

2Q: Two linked QTL

CEPH: Centre d'Etude du Polymorphisme Humain

chr: chromosome

GAW: Genetic Analysis Workshop

*h*^2^: heritability

MCMC: Markov chain Monte Carlo

MLE: Maximum likelihood estimate

QTL: Quantitative trait locus

SNP: Single-nucleotide polymorphism

VC: Variance components

## Competing interests

The author(s) declare that they have no competing interests.
